# Resident Use of Text Messaging for Patient Care: Ease of Use or Breach of Privacy?

**DOI:** 10.2196/medinform.4797

**Published:** 2015-11-26

**Authors:** Micah T Prochaska, Amber-Nicole Bird, Amar Chadaga, Vineet M Arora

**Affiliations:** ^1^ Section of Hospital Medicine Department of Medicine University of Chicago Chicago, IL United States; ^2^ General Internal Medicine Department of Medicine Perelman School of Medicine at University of Pennsylvania Philadelphia, PA United States; ^3^ Associate Program Director Internal Medicine Residency Program Advocate Christ Medical Center Chicago, IL United States; ^4^ Section of General Internal Medicine Department of Medicine University of Chicago Chicago, IL United States

**Keywords:** in-hospital communication, SMS text messaging, mobile technology

## Abstract

**Background:**

Short message service (SMS) text messaging is an efficient form of communication and pervasive in health care, but may not securely protect patient information. It is unclear if resident providers are aware of the security concerns of SMS text messaging when communicating about patient care.

**Objective:**

We sought to compare residents’ preferences for SMS text messaging compared with other forms of in-hospital communication when considering security versus ease of use.

**Methods:**

This study was a cross-sectional multi-institutional survey of internal medicine residents. Residents ranked different communication modalities based on efficiency, ease of use, and security using a Likert scale. Communication options included telephone, email, hospital paging, and SMS text messaging. Respondents also reported whether they had received confidential patient identifiers through any of these modalities.

**Results:**

SMS text messaging was preferred by 71.7% (94/131) of respondents because of its efficiency and by 79.8% (103/129) of respondents because of its ease of use. For security, 82.5% (104/126) of respondents preferred the hospital paging system, whereas only 20.6% (26/126) of respondents preferred SMS text messaging for secure communication. In all, 70.9% (93/131) of respondents reported having received patient identifiers (first and/or last name), 81.7% (107/131) reported receiving patient initials, and 50.4% (66/131) reported receiving a patient’s medical record number through SMS text messages.

**Conclusions:**

Residents prefer in-hospital communication through SMS text messaging because of its ease of use and efficiency. Despite security concerns, the majority of residents reported receiving confidential patient information through SMS text messaging. For providers, it is possible that the benefits of improved in-hospital communication with SMS text messaging and the presumed improvement in the coordination and delivery of patient care outweigh security concerns they may have. The tension between the security and convenience of SMS text messaging may represent an educational opportunity to ensure the compliance of mobile technology in the health care setting.

## Introduction

Mobile technology (mobile phones and tablets) has been shown to improve physician efficiency [1] and residents perceive it to improve inpatient communication [2-4]. Short message service (SMS) text messaging is one form of communication using mobile technology that is easy to use, accessible, and allows for the rapid and direct transfer of clinical information between providers. Therefore, SMS text messaging has become pervasive in health care [5] and is preferred for in-hospital communication between residents compared to a traditional in-hospital paging system [6]. Yet, SMS text messaging is discouraged by the Joint Commission for Healthcare Communication for security reasons [7] because there are serious concerns about its compliance with the US Health Insurance Portability and Accountability Act (HIPAA) and its ability to protect confidential patient health information when used on personal mobile devices [8].

Currently, it is unclear if the millennial generation of residents, who are comfortable with the ubiquity of SMS text messaging and its benefits, share the preceding concerns regarding SMS text messaging and patient confidentiality. Protecting patient confidentiality is a professional responsibility outlined in the ABIM Foundation physician charter on medical professionalism [9]. Examining residents’ understanding of if and how SMS text messaging may violate their obligation to patient confidentiality is one way of evaluating resident professionalism. Additionally, because behaviors learned and developed during medical training are often carried into future practice, it is particularly important to understand residents’ perceptions on the use of technology with respect to patient confidentiality [10,11].

Therefore, our study aimed to understand internal medicine residents’ preferences for SMS text messaging versus other available in-hospital communication modalities when considering efficiency, ease of use, and security. Additionally, we sought to determine residents’ experiences and perceptions of receiving confidential patient information through SMS text messaging.

## Methods

A cross-sectional paper survey was administered to internal medicine residents at 2 academic medical centers, one community-based and the other university-based, during the 2013-2014 academic year. Surveys were passed out to individual residents and collected during morning report and noon conference on different days to ensure that all residents willing to participate had the opportunity. The 2 surveyed institutions maintain residency programs represented by equal numbers of males and females. The hospital paging system with telephone call back was the institutionally preferred and supported method of provider communication at both institutions and neither institution supported or endorsed any form of SMS text messaging (including secure text messaging apps). Residents at both institutions were provided institutional emails, iPads (Cupertino, CA) for use in patient care, and at one institution on-call residents were also provided with portable phones for communication. The survey asked residents to rank on a 4-point Likert scale (1 was most preferred and 4 was least preferred) their preferred form of communication when considering efficiency, the ease of use, and the security of the communication modality. Responses were then dichotomized to represent either “preferred” or “not preferred.” Communication options included telephone, email, alphanumeric text (hospital) paging system, and SMS text messaging. Respondents were also asked to report whether they had received confidential patient identifiers (name, patient initials, or medical record numbers) through any of the these modalities.

## Results

**Figure 1:**
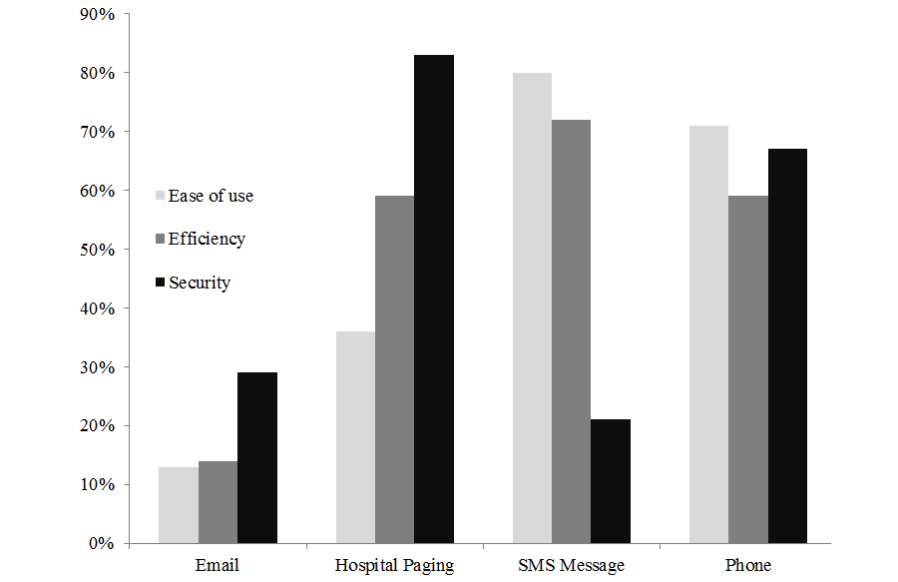
Preference for communication modality comparing ease of use, efficiency, and security.

**Figure 2:**
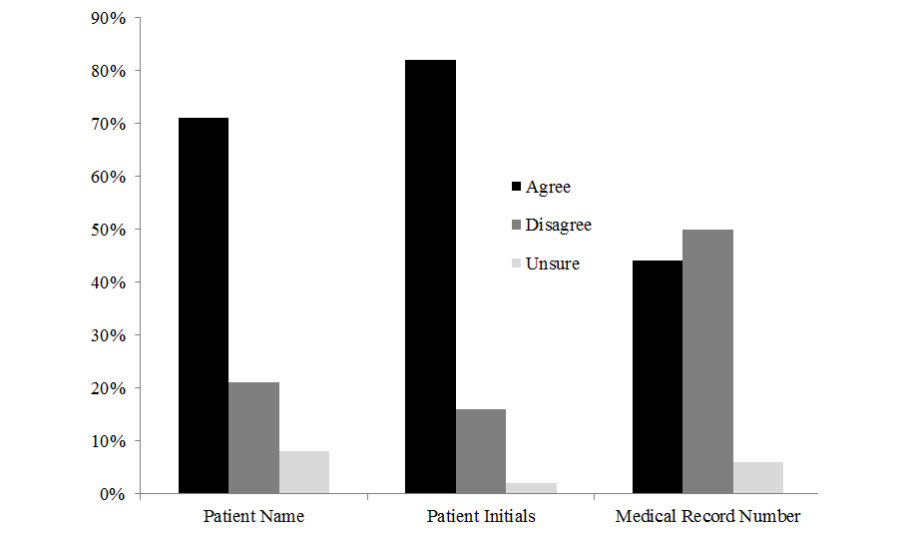
Received protected health information through SMS text messaging.

The overall response rate was 76.3% (132/173). For overall efficiency, 71.7% (94/131) of respondents preferred SMS text messaging, whereas 79.8% (103/129) of respondents reported SMS text messaging to be their preferred communication modality with respect to ease of use when communicating with other providers (Figure 1). In comparison, approximately one-third (35.6%, 46/129) of respondents preferred the current hospital paging system for ease of use when communicating with other providers. However, most (82.5%, 104/126) respondents rated the hospital paging system their preferred form of communication for security, whereas only 20.6% (26/126) of respondents preferred SMS text messaging for secure communication. Despite the security concerns of SMS text messaging, 70.9% (93/131) of respondents reported having received protected patient identifiers, including a patient’s first and/or last name, through SMS text messages (Figure 2). Many (81.7%, 107/131) reported receiving patient initials through SMS text messages and half (50.4%, 66/131) reported receiving a patient’s medical record number through SMS text messages. Responses did not vary by site.

## Discussion

Our data demonstrate that residents are aware of and concerned about the security of SMS text messaging, but prefer it for in-hospital communication because of its efficiency and ease of use. Despite these security concerns, a majority of residents reported receiving confidential patient information through SMS text messaging. One possible explanation for these results is that residents are faced with balancing the tradeoff between the presumed benefits of efficient and easier-to-use modes of in-hospital communication versus their belief about the security risk posed by communicating protected health information through the different available modes of communication. Interestingly, a majority of residents rated the hospital paging system their preferred method of communication with regards to security, although hospital pagers themselves are not HIPAA compliant [6]. The discrepancy in perception of the security risks of SMS text messaging compared to hospital paging may be due to an underappreciation of the risk of SMS text messaging and an overconfidence in the security of the paging system because it is institutionally supported by the hospital.

However, consequences exist if residents are individually balancing the tradeoff between the benefits of a technology such as SMS text messaging and the security risk it poses to protecting patient information. Residents or trainees may not be accurately estimating either the benefits of SMS text messaging or the real risks and consequences of a health care data breach [12,13]. Additionally, the pressure to be an efficient resident may cause some residents to utilize SMS text messaging in order to maximize efficiency despite the risks to patient confidentiality. In circumstances in which the use of SMS text messaging threatens confidentiality, it also threatens resident professionalism.

Therefore, this presents an educational opportunity to foster understanding about how HIPAA applies to new technologies such as SMS text messaging as well as to inform trainees about the true risks and consequences of data breaches involving protected health information [13]. HIPAA does not specifically ban SMS text messaging or other technologies, but it requires that any exchange of electronic health information meet the minimum standard for physical, network, and process security [14]. By not banning specific technologies, these expectations recognize the fact that new technologies can improve the efficiency and quality of care, but they require that providers and health systems together account for the rights of patients to have their information protected. Therefore, educators have a responsibility to help residents as frontline patient providers and not leave them isolated or at risk with the use of emerging technology. Rather, residents should receive formal education in the standards regarding technology and health care security. Additionally, they should also be engaged in finding and promoting technologies within their institutions, such as secure SMS text messaging apps that are both HIPAA compliant as well as efficient and easy to use. Lastly, residency program directors and institutions should strongly consider understanding the patterns of communication use among residents to ensure that resident practice is in-line with their hospital policy and that hospital policy supports technologies that are efficient, easy to use, and secure for communication between clinicians.

Our study is limited as a 2-institution study and it is possible that our results may not be generalizable to other institutions. Additionally, we collected self-reported data that may be subject to a socially desirable response bias. A socially desirable response bias would make respondents less likely to report having received confidential patient information through SMS text messages, which may mean our data underestimate the true frequency of this phenomenon. Additionally, our survey did not account for the possibility that resident communication preferences may vary based on which member of the medical team they are communicating with and that it is unlikely a resident could SMS text message another member of the medical team with whom they have had no previous contact.

We believe we are the first to study residents’ perception of the security of different communication modalities. Our findings suggest that although previous literature supports residents’ preference for SMS text messaging, residents are also aware of the security concerns of text messaging. However, the efficiency and ease of use of SMS text messaging when coordinating inpatient care may trump concerns that it does not adequately protect confidential patient information. The tension between the efficiency of a personal technology adapted into health care, but not designed to meets its security standards, will continue to arise as new technologies are developed. As the benefits of these technologies become manifest, we believe it is unrealistic to expect residents or other providers to abstain from their use or self-govern without proper continual education and institutional support to promote awareness of the complexities and nuances of technology and security in health care.

## Abbreviations

HIPAA: Health Insurance Portability and Accountability Act
SMS: short message service
